# Neurofascin (NF)155- and NF186-Specific T Cell Response in a Patient Developing a Central Pontocerebellar Demyelination after 10 Years of CIDP

**DOI:** 10.3389/fneur.2017.00724

**Published:** 2017-12-22

**Authors:** Juliane Klehmet, Max Staudt, Jan-Markus Diederich, Eberhard Siebert, Edgar Meinl, Lutz Harms, Andreas Meisel

**Affiliations:** ^1^Charité University Medicine Berlin, NeuroCure Clinical Research Center (NCRC), Berlin, Germany; ^2^Department of Neurology, Charité University Medicine Berlin, Berlin, Germany; ^3^Department of Neuroradiology, Charité University Medicine Berlin, Berlin, Germany; ^4^Institute of Clinical Neuroimmunology, University Hospital and Biomedical Center, Ludwig-Maximilians University of Munich, Munich, Germany

**Keywords:** CIDP, CCPD, neurofascin, neurofascin 155, atypical

## Abstract

**Background:**

Information and pathobiological understanding about central demyelinating manifestation in patients, who primarily suffer from chronic inflammatory demyelinating polyneuropathy (CIDP), are scarce.

**Methods:**

IFN-γ-response as well as antibodies against the (para)nodal antigens neurofascin (NF)155 and NF 186 had been tested by Elispot assay and ELISA before clinical manifestation and at follow-up.

**Case description and results:**

The patient described here developed a subacute brainstem syndrome more than 10 years after diagnosis of CIDP under low-dose maintenance treatment of intravenous immunoglobulins (IVIG). MRI revealed enhancing right-sided pontocerebellar lesion. CSF examination showed mild pleocytosis and elevated protein, and negative oligoclonal bands. Further diagnostics exclude differential diagnoses such as tuberculoma, sarcoidosis, or metastasis. Specific IFN-γ response against NF155 and NF186 as measured by Elispot assay was elevated before clinical manifestation. NF155 and NF186 antibodies were negative. Escalation of IVIG treatment at 2 g/kg BW followed by 1.4 g/kg BW led to clinical remission albeit to a new asymptomatic central lesion. Follow-up NF155 and NF186-Elispot turned negative.

**Conclusion:**

The case reported here with a delayed central manifestation after an initially typical CIDP and NF155 and NF186 T cell responses does not resemble described cases of combined central and peripheral demyelination but may reflect a novel subtype within the great clinical heterogeneity of CIDP.

## Introduction

Multiple sclerosis (MS) and chronic inflammatory demyelinating polyneuropathy (CIDP) are both demyelinating disorders of the nervous system with unknown underlying pathomechanisms. Whereas MS is restricted to the central nervous system, CIDP comprises demyelination of the peripheral nervous system. There are few reports in the literature about patients suffering from both, CNS and PNS manifestation in similar extent (1, 2). However, only little is known about central manifestations other than cranial nerve involvement over the course of disease in patients, who primarily suffer from CIDP. Due to its heterogeneous manifestation, different autoimmune targets are likely to be relevant in CIDP. T cell responses have been shown to be involved in its pathogenesis (3, 4).

## Methods

### ELISPOT

96-well plates (Millipore, Billerica, MA, USA) were coated with an IFN-γ-specific antibody (eBioscience, San Diego, CA, USA) at 4 µg/ml in sterile PBS overnight. After blocking with sterile PBS + 1% BSA (Sigma-Aldrich, St. Louis, MO, USA) for 60–120 min, fresh PBMCs were added in a number of 4 × 10^5^ cells/well in presence of anti-CD28 antibody (eBioscience) at 2 µg/ml. Peripheral myelin antigens neurofascin (NF)155 and NF186 [as described (5); kindly provided by E. Meinl, Munich] were added at 40 µg/ml. To detect spontaneous IFN-γ secretion CTL-Test-Medium (CTL, Cleveland, OH, USA) was used. CEF was used as positive control at a concentration of 10 µg/ml. It is a peptide pool containing 23 MCH class 1 restricted viral antigens (6). Plates were incubated at 37°C and 5% CO_2_ for 24 h. Mouse-antihuman IFN-γ biotin antibody (eBioscience) at concentration of 2 µg/ml conjugated to streptavidin-horseradish-peroxidase (BioLegend, San Diego, CA, USA) at 1:1,000 were used for detection. Plates were developed with 3-amino-9-ethyl carbazole reagent (Sigma-Aldrich, St Louis, MO, USA). The resulting spots were detected, counted, and analyzed *via* Elispot Reader (Autoimmun Diagnostika GmbH, Strassberg, Germany) and appendant iSpot 04 Software. Spot forming unit (SFU) for each antigen triplicate was averaged and subtracted by average SFU of spontaneous IFN-γ secretion and then calculated for a cell amount of 10^6^ cells.

### Patient Consent

The patient here gave her written consent as part of a cohort study before the study. She additionally gave her written informed consent for the publication of this case report. The cohort study was approved by ethical committee of Charité University Medicine Berlin. All patients were recruited in the outpatient clinic of Charité Department of Neurology. For Elispot assay control, we included 16 age-matched patients suffering from non-immune neuropathies.

### Description of the Case

The 56-year-old patient was diagnosed with CIDP in 1999 with a typical manifestation consisting of distal and proximal weakness and sensory dysfunction of all extremities. The clinical evaluation revealed generally absent deep tendon reflexes, distal and proximal weakness, large fiber sensory involvement, and a tremor of the right arm. Nerve conduction studies revealed primary demyelinating neuropathy (Table 1). She had suffered from tuberculosis infection 1965, 1974, and 1978. In 2002, she was diagnosed with breast cancer treated by tamoxifen.

**Table 1:** Nerve conduction studies before brainstem symptoms.

Nerve		DML	MCV	CMAP	F-wave
Median	Right	9 ms	34 m/s	3 mV	47 ms
	Left	10.8 ms	31 m/s	2 mV	51 ms
Ulnar	Right	8.2 ms	37 m/s	3.7 mV	51.4 ms
	Left	8.3 ms	35 m/s	3.2 mV	58 ms
Peroneal	Right	n.d.	n.d.	n.d.	n.d.
Tibial	Right	n.d	n.d	n.d	n.d

*DML, distal motor latency; MCV, motor nerve conduction; CMAP, compact motor action potential*.

**Figure 1:**
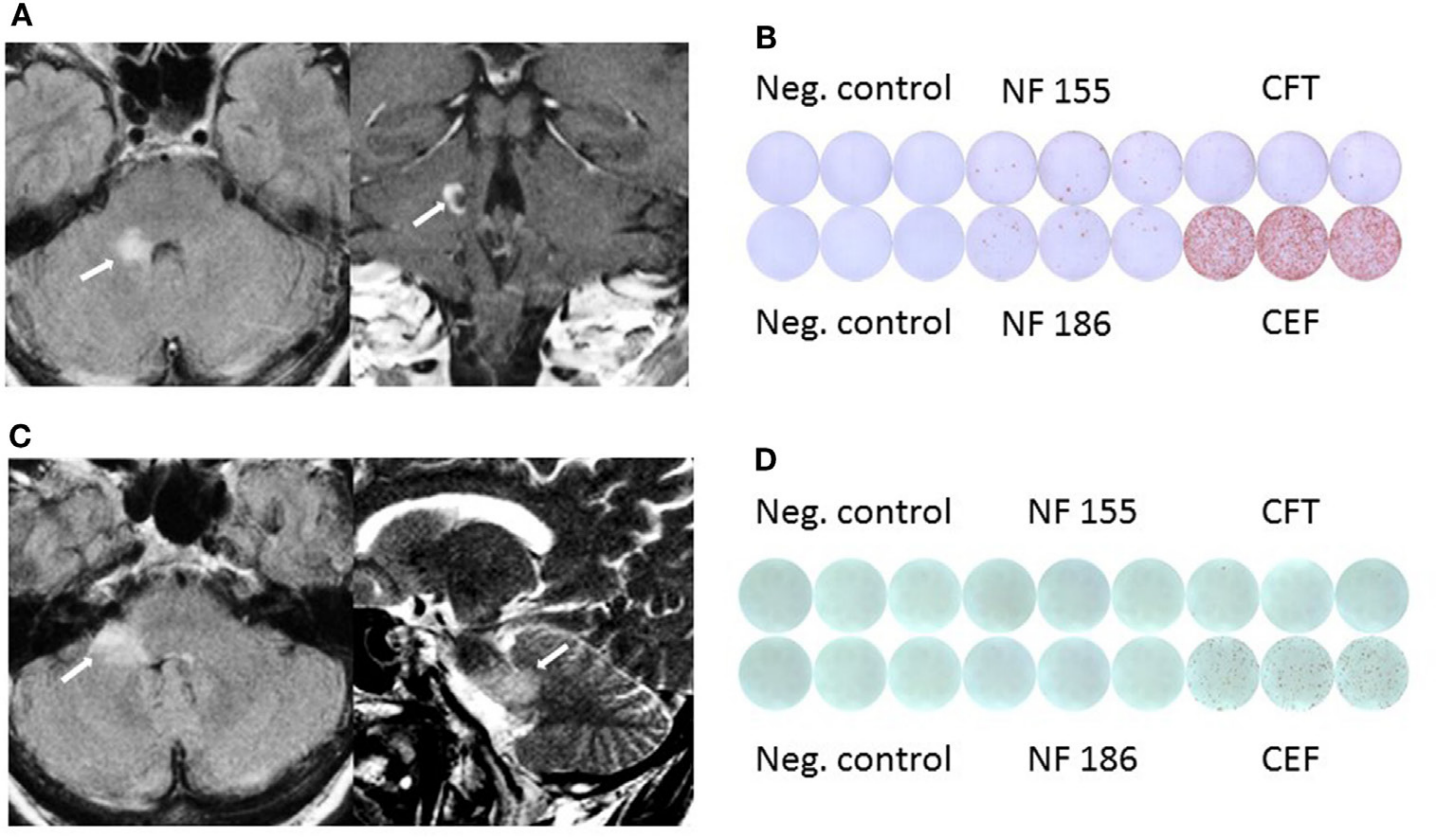
**(A)** MRI shows pontocerebellar lesion with incomplete peripheral enhancement. **(B)** Elevated IFNγ response against neurofascin (NF)155 and NF186 before clinical manifestation in Elispot assay. **(C)** Follow-up MRI shows new lesion in the middle cerebellar peduncle and adjacent pons without clinical manifestation or contrast enhancement. **(D)** Follow-up Elispot assay shows no IFNγ response against NF.

The patient received intravenous immunoglobulins (IVIG) treatment since 2004 with a dosage of 50 g at 4–10 weeks of treatment intervals (0.77 g/kg body weight) and was clinically stable for more than 10 years. In 2014, she subacutely developed progressive vertigo and a tendency to fall to the right side combined with nausea. In addition, she suffered from increasing problems with walking and climbing stairs. Clinical examination revealed a clear deterioration of the paresis but no nystagmus or other signs for vestibular involvement. Ear–nose–throat examination was without pathological finding. MRI demonstrated a right-sided pontocerebellar lesion adjacent to the upper cerebellar peduncle with incomplete peripheral enhancement. There was no concurrent pathological meningeal enhancement (Figure 1A).

As differential diagnosis tuberculoma, metastasis, sarcoidosis, MS, as well as central manifestation of CIDP were discussed and further diagnostic tests were performed. For that, CSF showed mild pleocytosis (seven cells) and elevated protein (136 mg/dl) with normal lactate levels, negative oligoclonal bands (OCB) without evidence for infection. *Borrelia* screening was negative. CT scan of the thorax showed two old, unchanged calcified granulomas in the left upper field of lung compatible with previous tuberculosis infection, there were no signs of lymphadenopathy. In addition, extensive tests revealed no sign for recurrence of breast carcinoma.

Importantly, before the patient developed the clinical manifestation of the brainstem symptoms we had demonstrated positive NF155- (mean 17.5 IFN-γ spots/10^6^ MNCs) as well as NF186-specific T cell response (28.33 IFN-γ spots/10^6^ MNCs) compared to CEF peptide pool specific IFN-γ response (1,062 IFN-γ spots/10^6^ MNCs) by ELISPOT assay (Figure 1B). In comparison, 16 patients with non-immune neuropathy revealed no NF155 (mean 0.5 IFN-γ spots/10^6^ MNCs) and NF186 (mean 0 IFN-γ spots/10^6^ MNCs) with CEF peptide pool specific IFN-γ response (519 IFN-γ spots/10^6^ MNCs). Interestingly, NF155 as well as NF186 antibodies measured by ELISA according to the protocol of Ng et al. (5) were negative.

In the synopsis of all findings, we diagnosed a subacute central manifestation of CIDP and escalated the immunomodulatory treatment by increasing the dosage of IVIG up to 2 g/kg body weight once followed by 1.4 g/kg BW. The patient recovered over 6 months reaching complete remission state of vertigo and improvement of gait ataxia.

Follow-up MRI scan 18 months later revealed a new, non-enhancing lesion within the middle cerebellar peduncle and the adjacent pons, the previously diagnosed lesion was not did no longer show gadolinium enhancement (Figure 1C). A follow-up Elispot assay did not show NF-specific T cell response any more (0 IFN-γ spots/10^6^ MNCs), while the IFN-γ response against the CEF control peptide pool was also reduced (mean 169 IFN-γ spots/10^6^ MNCs; Figure 1D). NF-specific antibodies remained negative. Follow-up CSF remained unchanged with a mild pleocytosis and elevated protein (129 mg/dl) without OCB.

## Discussion

There is still a controversy whether central manifestation in patients suffering from CIDP is a new entity of patients with combined central and peripheral demyelination (CCPD) or just a coincidence as further “subtype” among the line of atypical variants of CIDP. In addition, the underlying pathophysiology remains elusive. A recently published retrospective study of five patients described a pattern of extensive active demyelination of both, central and peripheral nervous system with insufficient treatment response with glucocorticosteroids (7). In a Japanese case study, patients suffering from CCPD showed a good response to IVIG (in 4/4 patients) or PE (in 2/3 patients) (8).

In a recently published retrospective study patients suffering from both CNS and PNS involvement (CCPD, *n* = 31) had a more heterogeneous clinical spectrum with frequent post-infectious onset, primary peripheral nervous system or central nervous system involvement, a monophasic or chronic disease course, inadequate response to treatments, and a generally poor outcome (2). 11 out of 31 CCPD patients had primary PNS involvement with GBS-like disease. PNS and CNS involvement was simultaneous in 22 patients (71%), CNS preceded PNS manifestation in 6 (19%) cases, whereas PNS preceded CNS involvement in 3 (10%) (2).

Due to the fact that the majority of the observed patients simultaneously or within a short interval affects central and peripheral nervous system, an autoimmune response directed against common epitopes which are presented in both CNS and PNS, is most likely. This autoreactive immune response can be mediated by autoreactive T-cells and/or autoantibodies. One of these common epitopes may be NF, which is expressed both in the CNS and PNS. Interestingly, antibodies directed against NF(155) had been described in a small subgroup of CCPD as well as in CIDP patients (9, 10). Anti-NF155 IgG4 antibodies had been found in patients suffering from CIDP (7%) associated with younger age at onset, ataxia, tremor, CNS demyelination (8%), and a poor response to IVIG (10). However, in another study, none of the patients with primarily PNS involvement had antibodies against NF (7) and similarly no anti-NF155 Abs were observed in CCPD (2, 11).

Similar to previous reports, our patient demonstrated extensive demyelination and suffered from sensory ataxia and tremor. However, in contrast to the majority of previous case reports, our patient developed the central manifestation only several years after onset of PNS demyelinating disease, which remained stable under IVIG-maintenance treatment. Interestingly, escalation of IVIG treatment was associated with a remission of clinical symptoms due to the central manifestation suggesting an insufficient IVIG dosage before. Most interestingly, we found NF155- and NF186-specific IFN-γ T cell responses together with an activated T cell reactivity as measured by CEF-specific IFN-γ response before clinical onset of brainstem manifestation. Interestingly, in a cohort study of typical and atypical CIDP patients, we found NF155- and NF186-specific T cell response in some patients but no specific antibody response (in submission). At the time of clinical remission and after high-dosage IVIG treatment, NF-specific T cell responses turned negative in the peripheral blood, which may support the role of NF as a pathogenically relevant epitope in CCPD. As we have demonstrated previously efficient IVIG treatment leads to reduction of CD8+ effector memory T cells (3), which may have led to reduced NF and thus clinical remission in the presented case.

In summary, the clinical presentation of CIDP with a central manifestation upon immunomodulatory treatment a decade after disease onset differs from so-far described CCPD cases and may present a novel subtype of CIDP. For the first time, we found a NF155- and NF186-specific T cell response before clinical onset of a central manifestation in CIDP patients. NF may therefore function as a specific antigen capable to elicit T and/or B cell autoreactive response in peripheral and central demyelinating disease.

## Ethics Statement

The patient here gave her written consent as part of a cohort study before the study. The cohort study was approved by ethical committee of Charité University Medicine Berlin. This study was carried out in accordance with the recommendations of ethical committee Charité University Medicine Berlin with written informed consent from all subjects. All subjects gave written informed consent in accordance with the Declaration of Helsinki. The protocol was approved by the by ethical committee of Charité University Medicine Berlin. All patients were recruited in the outpatient clinic of Charité Department of Neurology. For Elispot assay control, we included 16 age-matched patients suffering from non-immune neuropathies.

## Author Contributions

JK: design of the study, analysis of the data, and drafting the manuscript. MS, JD and ES: acquisition and collecting of data, analysis of data. EM, LH, and AM: revising the manuscript for intellectual content, interpretation of pathological data.

## Conflict of Interest Statement

JK reports grants from Grifols, grants from German Research Foundation during the conduct of the study; personal fees from personal compensation for speaker fees and advisory boards outside the submitted work from Grifols, Octapharma, and CSL Behring, outside the submitted work. MS, J-MD, and ES have nothing to disclose. EM reports grant support from Novartis and personal compensations from Genzyme and Roche outside the submitted work. LH reports personal fees from TEVA, personal fees from Bayer, grants from Merck, grants and personal fees from Biogen, grants and personal fees from Roche, personal fees from Novartis, personal fees from Genzyme, grants from Grifols, outside the submitted work. AM reports grants from Grifols, during the conduct of the study; personal fees from Grifols, personal fees from Octapharma, personal fees from CSL Behring, outside the submitted work. The reviewer EM and handling editor declared their shared affiliation.
